# Supplementary material for: Explaining the increase of incidence and mortality from cardiovascular disease in Indonesia: A global burden of disease study analysis (2000–2019)
Source: PLoS One. 2023 Dec 15;18(12):e0294128. doi: 10.1371/journal.pone.0294128 (PMC10723707; doi:10.1371/journal.pone.0294128)
Supplement: S1 File — (DOCX) [file pone.0294128.s001.docx]

**Supplementary Table 1:** Descriptive information on factors and data sources utilized in Indonesia's 34 provinces from 2000 to 2019.

| Variable Name | Unit | Source | Date Last Accessed |
| --- | --- | --- | --- |
|  |  | **Socioeconomic indicators** |  |
| GDP per capita | Rupiah (Rp.) | Gross Regional Domestic Product of Provinces in Indonesia by Industrial Origin. BPS-Statistics Indonesia. Available at:  https://bps.go.id/searchengine/result | 15 ^th^ May 2023 |
| Coverage of Primary care | (n) per 100 people | Indonesia Health Profile. Ministry of Health, Indonesia. Available at:  https://kemkes.go.id/folder/view/01/structure-publikasi-pusdatin-profil-kesehatan | 15 ^th^ May 2023 |
| Socio-demographic Index | 0-1 | Global Health Data Exchange/GBD Results Tool. Available at: https://vizhub.healthdata.org/gbd-results/ | 15 ^th^ May 2023 |
|  |  | **Health outcomes and risks** |  |
| Mortality Rate | Death per 100.000,00 | Global Health Data Exchange/GBD Results Tool. Available at: https://vizhub.healthdata.org/gbd-results/ | 15 ^th^ May 2023 |
| Summary Exposure Value | Risk-adjusted prevalence, from 0 to 1 | Global Health Data Exchange/GBD Results Tool. Available at: https://vizhub.healthdata.org/gbd-results/ | 15 ^th^ May 2023 |
| Population attributable fraction (PAF) | % | Global Health Data Exchange/GBD Results Tool. Available at: https://vizhub.healthdata.org/gbd-results/ | 15 ^th^ May 2023 |

**Supplementary Table 2:** : International Classification of Diseases (ICD) codes and hierarchy for non-communicable diseases (NCDs) included in the analysis.

| **Metric** | **ICD-10** | **ICD-9** |
| --- | --- | --- |
| Cardiovascular Diseases | B33·2, G45-G46·8, I01-I01·9, I02·0, I05-I09·9, I11-I11·9, I20-I25·9, I28-I28·8, I30-I31·1, I31·8-I37·8, I38-I41·9, I42·1-I42·8, I43-I43·9, I47-I48·9, I51·0-I51·4, I60-I63·9, I65-I66·9, I67·0-I67·3, I67·5-I67·6, I68·0-I68·2, I69·0-I69·3, I70·2-I70·8, I71-I73·9, I77-I83·9, I86-I89·0, I89·9, I98, K75·1 | 036·4, 391-391·9, 392·0, 393-398·9, 402-402·9, 410-414·9, 417-417·9, 420-423, 423·1-423·9, 424·0-424·9, 425·0-425·3, 425·5, 425·7-425·8, 427-427·3, 427·6-427·8, 429·0, 430-435·9, 437·0-437·2, 437·5-437·8, 440·2, 440·4, 441-443·9, 447-454·9, 456, 456·3-457, 457·1, 457·8-457·9, 459, 459·1-459·3 |
| Ischaemic Heart Disease | I20-I25·9 | 410-414·9 |
| Ischemic Stroke | G45-G46·8, I63-I63·9, I65-I66·9, I67·2-I67·3, I67·5-I67·6, I69·3 | 433-435·9, 437·0-437·1, 437·5-437·8 |
| Diabetes Mellitus Type 2 | E11-E11·1, E11·3-E11·9 |  |

ICD is the global standard for health data, clinical documentation, and statistical aggregation. Abbreviations: DE= Disease endpoint

**Supplementary Table 3:** Analytical metadata for risk factors.

| **Metric** | **Definition** | **Scores** |
| --- | --- | --- |
| **High Body-mass Index** | Serum fasting plasma glucose measured in mmol/L | 20–25 kg/m² |
| **High Systolic Blood pressure** | Systolic blood pressure, measured in mmHg | 110–115 mm Hg |
| **High fasting plasma glucose** | Body-mass index, measured in kg/m² | 4.8–5.4 mmol/L |
| **Hyperlipidemia** | High LDL cholesterols, measured in mmol | 1.3 mmol/L |
| **Smoking** | Prevalence of present use of any smoked tobacco product and prevalence of previous use of any smoked tobacco product; cigarette equivalents smoked per smoker per day and cumulative pack-years of exposure among current smokers; number of years since quitting among former smokers | All individuals are lifelong non-smokers |

**Supplementary Table 4**

| **Metric** | **Definitions** |
| --- | --- |
| **Summary Exposure Value (SEV)** | A measure of a population's exposure to risk factors that takes into account both the extent of exposure by risk level and the severity of the risk's contribution to disease burden. SEV has a value of zero when there is no additional risk for a population and a value of one when the entire population is at the highest risk; we portray SEV on a scale ranging from 0% to 100% to indicate that it is risk-weighted prevalence. |
| **Population Attributable Fraction (PAF)** | The population attributable fraction is the proportional reduction in mortality that would occur in a population if the risk factor exposure were lowered to an optimum exposure situation. The number of deaths attributable to a risk factor is calculated by dividing the total number of deaths by the population attributable fraction. |
| **Primary Health Care** | Primary Health Care (Puskesmas) is the first level of health care and is defined by a set of health actions at the individual and collective levels that include health promotion and protection, disease prevention, diagnosis, treatment, rehabilitation, harm reduction, and health maintenance to develop comprehensive care that has a positive impact on community health. |
| **GDP percapita** | GDP per capita is calculated by dividing gross domestic product by the midyear population. GDP is calculated as the sum of the gross value added by all resident producers in the economy, plus any product taxes and minus any subsidies not included in the product value. It is computed without regard for depreciation of manufactured assets or depletion and deterioration of natural resources. |
| **Sociodemographic Index** | The Socio-demographic Index (SDI) is a composite metric that gives a snapshot of a country's social and economic development. The Global Burden of Disease (GBD) research created it to classify countries depending on their level of development. To assess a country's overall socio-demographic status, the SDI integrates many indices relating to income, education, and fertility rate. |
